# The Application of Nanomaterials in Breast Cancer

**DOI:** 10.3390/pharmaceutics17121608

**Published:** 2025-12-14

**Authors:** Kexin Guo, Yue Sun, Huihua Xiong

**Affiliations:** Department of Oncology, Tongji Hospital, Tongji Medical College, Huazhong University of Science and Technology, Wuhan 430030, China; m202476582@hust.edu.cn

**Keywords:** breast cancer, nanomaterials, cancer therapy, theranostic

## Abstract

Breast cancer is one of the most prevalent malignant tumors worldwide, with the highest incidence and mortality among women. Early precise diagnosis and the development of efficient treatment regimens remain major clinical challenges. Harnessing the programmable size, surface chemistry, and tumor microenvironment (TME) responsiveness of nanomaterials, there is tremendous potential for their applications in breast cancer diagnosis and therapy. In the diagnostic arena, nanomaterials serve as core components of novel contrast agents (e.g., gold nanorods, quantum dots, superparamagnetic iron oxide nanoparticles) and biosensing platforms, substantially enhancing the sensitivity and specificity of molecular imaging modalities—such as magnetic resonance imaging (MRI), computed tomography (CT), and fluorescence imaging (FLI)—and enabling high-sensitivity detection of circulating tumor cells and tumor-derived exosomes, among various liquid biopsy biomarkers. In therapy, nanoscale carriers (e.g., liposomes, polymeric micelles) improve tumor targeting and accumulation efficiency through passive and active targeting strategies, thereby augmenting anticancer efficacy while effectively reducing systemic toxicity. Furthermore, nanotechnology has spurred the rapid advancement of emerging modalities, including photothermal therapy (PTT), photodynamic therapy (PDT), and immunotherapy. Notably, the construction of theranostic platforms that integrate diagnostic and therapeutic units within a single nanosystem enables in vivo, real-time visualization of drug delivery, treatment monitoring, and therapeutic response feedback, providing a powerful toolkit for advancing breast cancer toward personalized, precision medicine. Despite challenges that remain before clinical translation—such as biocompatibility, scalable manufacturing, and standardized evaluation—nanomaterials are undoubtedly reshaping the paradigm of breast cancer diagnosis and treatment.

## 1. Introduction

Breast cancer stands as the most commonly diagnosed malignancy and the leading cause of cancer-related mortality among women worldwide, posing a significant challenge to global public health. According to the latest global cancer statistics released by the International Agency for Research on Cancer (IARC) in 2022, there were over 2.3 million new cases of breast cancer and approximately 670,000 deaths, with a concerning trend towards earlier onset age, highlighting the increasing urgency of prevention and treatment [1]. Currently, imaging techniques such as mammography, ultrasound, and MRI serve as the cornerstone for breast cancer screening and diagnosis [2]. However, their diagnostic accuracy is often limited by factors like dense breast tissue and tumor heterogeneity, making it difficult to achieve early detection at the molecular level [3,4]. In terms of treatment, comprehensive therapeutic strategies based on surgery, radiotherapy, chemotherapy, endocrine therapy, and targeted drugs are widely used but continue to face numerous challenges, including multidrug resistance, significant systemic toxicity, and high rates of metastasis and recurrence [5]. A more profound challenge stems from the high degree of molecular heterogeneity in breast cancer—categorized into subtypes such as Luminal A, Luminal B, human epidermal growth factor receptor 2 (HER2)-positive, and triple-negative based on distinct molecular marker profiles [6]—which renders the single therapeutic strategy approach inadequate for meeting the pressing demands of personalized medicine. Consequently, the development of novel technological platforms capable of simultaneously achieving highly sensitive diagnosis and highly effective, low-toxicity treatment has emerged as a critical scientific problem to overcome the current dilemmas in breast cancer management.

The rapid advancement of nanotechnology offers unprecedented opportunities to address these challenges [7,8,9,10,11,12]. Nanomaterials, leveraging their unique size effects, engineerable surfaces, multimodal compatibility, and intelligent responsiveness to the TME, can function as “precision-guided missiles,” enabling active targeting and accumulation at tumor sites within the body [7]. This significantly enhances the delivery efficiency and therapeutic index of conventional chemotherapeutic agents, nucleic acid-based drugs, photosensitizers (PSs), and immunomodulators [11,12]. On the diagnostic front, through the conjugation of specific targeting moieties (such as antibodies or ligands targeting HER2, estrogen receptor (ER), protein ligand-1 (PD-L1), etc.), nanoprobes can achieve highly sensitive recognition of breast cancer-associated molecular biomarkers, providing powerful tools for early disease detection, intraoperative navigation, and dynamic therapeutic monitoring [9,10]. In this context, the construction of “theranostic” nanoplatforms is particularly noteworthy. By integrating diagnostic, therapeutic, and treatment evaluation functions into a single nanosystem, they open up a new paradigm for the personalized, visualized, and real-time management of breast cancer [13].

This review systematically summarizes the latest advances of nanomaterials in the diagnosis and treatment of breast cancer. It begins by outlining the applications of nanomaterials in breast cancer biomedical imaging and biomarker detection. It then assesses cutting-edge developments in areas such as intelligent drug delivery, immunotherapy, gene therapy, and phototherapy. Finally, it highlights strategies for constructing integrated theranostic nanoplatforms and discusses their prospects for clinical translation. This work aims to provide theoretical guidance and practical directions for advancing nanomedicine toward precision diagnosis and therapy of breast cancer.

## 2. Nanomaterials for Diagnosis in Breast Cancer

**Figure 1:**
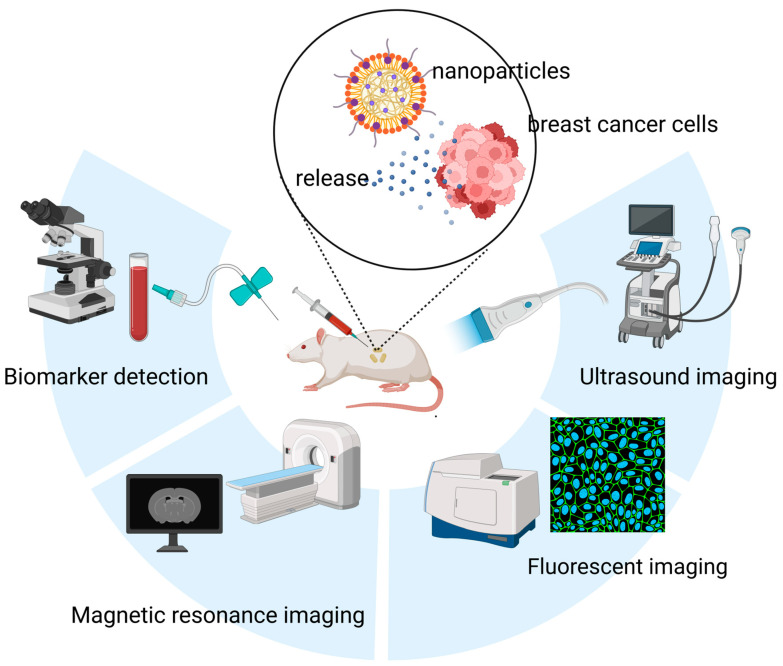
Applications of nanomaterials in the diagnosis of breast cancer, including nanomaterials-based in vitro detection techniques, and nanomaterials-enabled in vivo imaging techniques (e.g., Ultrasound (US) imaging, FLI and MRI).

Conventional imaging modalities face inherent limitations in sensitivity, often failing to detect breast microcalcifications or to define molecular-level margins [14,15]. This shortfall contributes to high rates of positive margins following breast-conserving surgery and the oversight of micrometastases in sentinel lymph nodes [15,16]. Furthermore, cancer development and progression are closely associated with alterations in the expression and concentration of various biomarkers, including cell-surface proteins, regulatory genes, and signaling molecules. In breast cancer, conventional diagnostic approaches often rely on the detection of protein biomarkers such as Carbohydrate antigen 15-3 (CA15-3) and Carcinoembryonic Antigen (CEA), typically through antibody- or aptamer-based assays [17]. Additionally, certain microRNAs (miRNAs) have emerged as promising diagnostic molecules, though their clinical application is often hindered by complex and costly detection procedures [18]. In this context, nanomaterials have gained considerable attention due to their high sensitivity, ease of use, and cost-effectiveness, offering new avenues for breast cancer diagnostics (Figure 1).

### 2.1. Imaging Nanoprobes

Nanoprobes, by virtue of their programmable surfaces, inherent signal amplification capabilities, and responsiveness to the TME, offer a transformative potential. They enable the detection of early lesions at a molecular scale, facilitate real-time intraoperative delineation of tumor boundaries, and provide concurrent insights into therapeutic response [19].

As a cornerstone of breast cancer management, MRI plays a pivotal role in screening high-risk individuals, assessing the extent of metastasis, and detecting local recurrence [20]. Magnetic nanoparticles can influence signal intensity by altering longitudinal (T1) and transverse (T2) relaxation times, making them highly promising as contrast agents [21]. Khaniabadi et al. developed superparamagnetic iron oxide nanoparticles (SPIONs) conjugated with the monoclonal antibody C595, enabling selective targeting of the breast-specific membrane antigen. Their results showed that the T2 relaxivity of the highest dose of SPIONs–C595 was markedly reduced, producing negative contrast in T2-weighted MRI [22]. Riboflavin (Rf) derivatives can serve as drug delivery ligands and bind to riboflavin carrier protein (RCP), which is overexpressed in breast cancer tissues [23]. Mekseriwattana et al. designed Rf-SPION based on Rf, and the results indicated that SPIONs exhibit a high r2 relaxivity of 430 mM^−1^ s^−1^ in water. In addition to SPIONs, nanoparticles containing gadolinium (III) (Gd^3+^) and manganese (II) (Mn^2+^) have shown promise in MR molecular imaging [24,25]. Han et al. designed a Gd^3+^-based nanoparticle conjugated with the ZD2 peptide, which targets extra domain-B fibronectin (EDB-FN), a marker of epithelial–mesenchymal transition (EMT). Their findings confirmed that even at low doses, this nanoparticle maintains high sensitivity and exceptional resolution for rapidly proliferating, invasive TNBC [26].

In recent years, FLI in the second near-infrared (NIR-II) window has attracted attention in cancer imaging due to its substantially reduced photon absorption and scattering in biological tissues, enhanced deep-tissue penetration, and high signal-to-noise ratio. Poly(sarcosine)-type polymers (PSar) are attractive candidates for NIR-II contrast agents because of their good aqueous solubility and intrinsic degradability [27]. Chen and colleagues designed PSar-modified nanoparticles (TQF–PSar) for imaging breast cancer lung metastasis. Compared with traditionally PEGylated nanoparticles, TQF–PSar-modified nanoparticles exhibit effective accumulation in tumor tissue, higher NIR-II fluorescence intensity, and longer circulatory half-life [28]. What’s more, to improve the accumulation efficiency of indocyanine green (ICG) in tumor tissues and enhance imaging performance, numerous ICG-based nanoprobe systems have been developed with promising results [29]. Yang et al. developed a biodegradable cyclic RGD pentapeptide/hollow virus-like Gd-based ICG nanoprobe (R&HV-Gd@ICG). Experimental results demonstrated that R&HV-Gd@ICG can accurately distinguish tumor tissue from healthy tissue during surgery, and its combination with radiotherapy further enhances sensitivity [30]. Furthermore, NIR nanomaterials demonstrate significant potential in breast cancer diagnosis and treatment. Their exceptional real-time imaging capabilities unlock the potential for surgical navigation and visualization of therapeutic response, thereby significantly advancing the construction of integrated diagnostic and therapeutic platforms, which will be elaborated in the following sections.

Aberrant glycosylation is a recognized hallmark of cancer, characterized by the overexpression of specific carbohydrate structures—such as Tn antigen and sialyl Lewis X antigen—on the cell surface. Lectins, natural proteins that bind carbohydrates with high specificity, have emerged as valuable tools for detecting these tumor-associated glycan patterns [31]. Incorporating lectins into nanoprobes represents a promising strategy to enhance the targeting and specificity of diagnostic platforms. For example, Cramoll 1,4 lectin, which recognizes overexpressed glucose/mannose residues on breast cancer cells, has been employed to functionalize quantum dots, improving both the sensitivity and contrast of fluorescence imaging [32]. The mannose receptor (MR), a lectin receptor overexpressed on the surface of breast cancer cells, offers another targeting opportunity [33]. Sha et al. developed a fluorescent probe by conjugating mannose to protein-coated gold nanoclusters for the detection of Concanavalin A (Con A) and imaging of breast cancer cells. The resulting sensor demonstrated high selectivity and sensitivity toward Con A, while the specific mannose-MR interaction enabled selective imaging of breast cancer cells [34].

In addition to the aforementioned nanoprobes, dual- or multi-modal imaging probes are increasingly being applied for early diagnosis of breast cancer. Mahdavimehr and colleagues functionalized SPIONs with diethylenetriaminepentaacetic acid (DTPA) and a novel C-peptide, which not only reduced toxicity but also provided functional groups for conjugating targeting ligands and chelating radiometals, thereby enhancing dual-modal imaging capabilities. MRI results showed significant contrast enhancement in tumor tissue 1.5 h after injection of SPION-DTPA-Pep, and single-photon emission computed tomography (SPECT) demonstrated substantial accumulation of SPION-DTPA-Pep-^99m^Tc in tumor tissue compared with free ^99m^Tc [35]. Jain et al. synthesized Gd_2_O_3_: Eu^3^+ nanoparticles, conjugated them with folic acid (FA), and evaluated their functionality using fluorescence and CT imaging. The results revealed strong red emission is exhibited at 613.4 nm within tumor tissue, and CT imaging indicated efficient accumulation of the nanoparticles in the tumor [36].

In summary, imaging nanoprobes are revolutionizing breast cancer diagnosis and management by enabling molecular-level detection of early lesions and micrometastases, providing real-time, precise intraoperative delineation of tumor margins, and allowing dynamic monitoring of therapeutic response—collectively advancing the frontier of precision oncology.

### 2.2. Nanomaterials in Biomarker Detection

Biomarkers serve as pivotal tools in the management of breast cancer, offering not only deep insights into the cellular and molecular pathways underlying the disease pathogenesis but also providing direct guidance for formulating treatment strategies. As a key driver protein in breast cancer, HER2 regulates cell growth, differentiation, and apoptosis by mediating complex signal transduction networks. Given that the HER2-positive subtype is generally associated with heightened aggressiveness and poorer clinical outcomes, the early and accurate determination of HER2 status has become pivotal for clinical decision-making [37,38]. To this end, nanomaterials have been leveraged in the development of HER2 detection technologies by virtue of their unique advantages, thereby offering novel approaches for highly efficient and sensitive diagnosis. For instance, Xu et al. constructed a tetrahedral nanostructure with dual specificity for HER2 and CD63, enabling sensitive detection of HER2-positive EVs. This platform demonstrated diagnostic capability for HER2-positive breast cancer at a threshold of 4.219 U·μL^−1^, offering a novel tool for noninvasive subtyping [39]. Besides, Shahbazi et al. designed a plasmonic biosensor based on the localized surface plasmon resonance (LSPR) of gold nanoparticles (AuNPs) for highly sensitive detection of HER2. The AuNP-based sensor exhibits an order of magnitude higher sensitivity than ELISA (10^−7^ M vs. 10^−6^ M) [40], and the detection limit is 3.7 × 10^−9^ M [41].

CA15-3 serves as an important biomarker for breast cancer monitoring and is widely used in the surveillance of metastatic disease [42]. However, conventional detection methods—such as enzyme-linked immunosorbent assay (ELISA), electrochemical immunoassay, and electrochemiluminescence immunoassay—suffer from limitations including long processing time, limited sensitivity, and operational complexity [43]. Therefore, the development of novel detection techniques holds significant importance for improving early diagnosis and prognostic evaluation of breast cancer. Pourmadadi et al. developed an electrochemical aptasensor for CA15-3 detection, which demonstrated superior sensitivity compared to conventional methods, achieving a detection limit of 0.2 U·mL^−1^ [44]. Chen et al. developed a label-free electrochemical immunosensor utilizing gold nanoparticles, which demonstrated a wide detection range (0.1–300 U·mL^−1^) and a low detection limit (0.011 U·mL^−1^) for CA15-3 detection [45]. In a separate study, Li and colleagues fabricated a multi-channel dual-gated silicon nanowire field-effect transistor (SiNW-FET) biosensor capable of simultaneously detecting CA15-3 and CEA with high sensitivity and specificity, offering a promising clinical technology for early breast cancer diagnosis and therapeutic monitoring [46]. Notably, CA15-3 is not only upregulated in breast cancer but also displays distinct alterations in its glycosylation profile [47]. To exploit this feature, Joonas et al. developed a lectin-nanoparticle-based assay for profiling the glycosylation of CA15-3. Among various lectin-Eu^3+^-nanoparticle conjugates tested, wheat germ agglutinin (WGA)-modified Eu^3+^-nanoparticles exhibited superior diagnostic performance in metastatic breast cancer patients, achieving a sensitivity of approximately 81.1% at 90% specificity—significantly higher than the conventional CA15-3 immunoassay with a sensitivity of 66.0% [48].

Liquid biopsy, an emerging diagnostic and detection technology, is gaining increasing utility in the analysis of cancer prognosis and metastatic progression. By detecting circulating biomarkers in body fluids—such as tumor cells, extracellular vesicles (EVs), proteins, and miRNAs—this approach overcomes the spatiotemporal limitations of traditional tissue biopsies, allowing noninvasive access to systemic physiological and pathological information [49,50]. The integration of electrochemical detection (ECD) methods with nanostructured electrodes has achieved notable progress in the field of breast cancer liquid biopsy, substantially enhanced the analytical performance of detection. Circulating tumor DNA (ctDNA) also represents a valuable biomarker in liquid biopsy. Due to its short half-life, ctDNA levels in blood can change rapidly, reflecting real-time dynamics in metastatic tumor burden [51,52,53]. Leveraging this feature, Park et al. integrated Fe_3_O_4_–Au core–shell nanoparticles with PCR in an electrochemical detection system, enabling sensitive (3 aM) and rapid (within 7 min) identification of metastatic breast cancer-derived ctDNA [54]. In addition to ctDNA, miRNAs have also been widely investigated due to their differential expression in cancer tissues and roles in regulating physiological and pathological processes [55]. For example, miR-155 is significantly upregulated in the serum of breast cancer patients and decreases following surgery or chemotherapy, supporting its utility as a diagnostic and monitoring biomarker [56]. Azimzadeh and colleagues developed a nano-biosensor using gold nanorods (GNRs) and graphene oxide (GO) for miR-155 detection, achieving a detection limit of 0.6 fM and a dynamic range from 2.0 fM to 8.0 pM [57]. In a separate study, Sadrabadi et al. constructed an electrochemical biosensor based on functionalized metal–organic frameworks (MOFs) and carbon nanostructures, using hematoxylin as an electrochemical label to recognize double-stranded RNA. This design achieved an ultralow detection limit of 0.08 fM for miR-155, with a broad dynamic range spanning 0.2 fM to 500 pM [58]. Another miRNA of interest, miR-21, is also dysregulated in breast cancer and has diagnostic potential [59]. Salahandish et al. designed a nanobiosensor capable of detecting miRNA across a wide dynamic range (10 fM to 10 μM), highlighting its potential for early breast cancer diagnosis [60].

In summary, nanomaterials have markedly improved the detection of breast cancer biomarkers by enabling the development of highly sensitive, specific, and user-friendly biosensors. Driven by nanotechnology, these platforms effectively address the limitations of conventional assays, especially when coupled with the liquid biopsy approach. Together, they pave a promising new path toward noninvasive early detection, molecular subtyping, real-time therapeutic monitoring, and accurate prognosis in breast cancer management (Table 1).

## 3. Nanomaterials for Therapy in Breast Cancer

**Figure 2:**
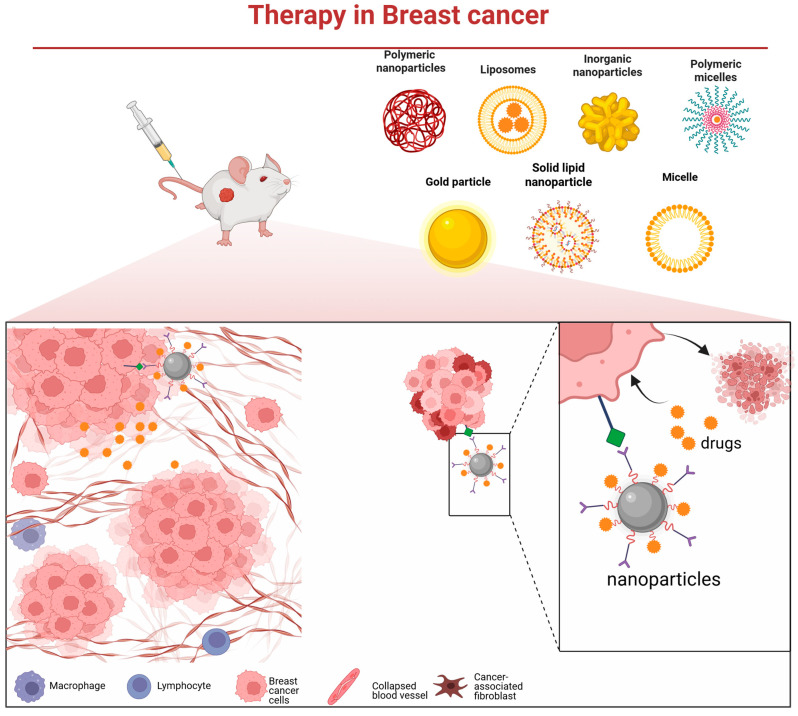
Various nanocarrier platforms utilized in breast cancer therapy and their operational mechanism—they bind to cancer cells via specific receptors, subsequently release the therapeutic payload, and thereby induce tumor cell death.

The pronounced molecular heterogeneity of breast cancer underpins the markedly divergent responses to chemotherapy and clinical outcomes observed among its subtypes [63]. While chemotherapy remains a cornerstone of adjuvant treatment, the development of drug resistance continues to limit its efficacy. There is, therefore, an urgent need for novel and precision-guided therapeutic strategies. Nanodelivery systems offer a promising platform to address this challenge [64]. They enable the targeted delivery of high-dose chemotherapeutics, molecularly targeted agents, nucleic acids, or immunomodulators deep into tumor tissues (Figure 2), while minimizing off-target effects [65]. Furthermore, such systems can be integrated with photothermal, photodynamic, or radiosensitizing modalities to empower multimodal synergistic therapy.

### 3.1. Chemotherapy Drug Delivery System

Several nano-formulated chemotherapeutic agents, including liposomal doxorubicin (DOX), have already received clinical approval for breast cancer treatment. The DOX/Cyclophosphamide (AC) regimen represents a classical therapeutic approach associated with high survival rates; however, its utility is limited by common adverse effects—such as cardiotoxicity, hepatotoxicity, and secondary leukemia—as well as the frequent development of acquired resistance [66,67]. These challenges underscore the need for more efficient and tumor-selective drug delivery strategies (Table 2). Poly (lactic-co-glycolic acid) (PLGA), as a biocompatible and biodegradable polymer, has been employed for the delivery of various anti-tumor drug [68]. To improve the therapeutic profile of DOX, Helmy et al. developed folate receptor-targeted PLGA nanoparticles co-loaded with DOX and trans-ferulic acid (TFA), a natural compound with anticancer activity but poor aqueous solubility [69]. The resulting DOX/FA-PLGA-TFA NPs not only reduced adverse effects relative to the free drug regimen but also demonstrated robust antitumor efficacy and an improved safety profile [70]. In a related effort, Hu et al. functionalized PLGA nanoparticles with tannic acid and Fe (III) ions to construct a stimuli-responsive delivery platform for DOX. This system promoted enhanced drug release under acidic conditions, improved cellular uptake in breast cancer cells, and achieved a notable tumor inhibition rate of 85.53 ± 8.77% [71]. Similarly, Brzeziński et al. developed tannic acid- and DOX-conjugated nanoparticles based on PCL/PTMC polymers, which suppressed tumor cell proliferation more effectively than free DOX [72].

Platinum-based agents such as cisplatin (CDDP) remain first-line chemotherapeutics that induce apoptosis through DNA damage [78]. Recent efforts have focused on nano-delivery systems to optimize the pharmacokinetics and tumor accumulation of CDDP. For example, Xiang et al. encapsulated cisplatin into core–shell nanoparticles stabilized by polyphenol–metal coordination. The resulting Polyethylene glycol (PEG)-GAx/Pt nanoparticles exhibited dual pH- and reactive oxygen species (ROS)-responsive drug release, leading to enhanced antitumor activity and reduced systemic toxicity [73]. In another study, Sultan et al. developed cisplatin-loaded chitosan nanoparticles (CCNP), which effectively induced early and late apoptosis along with chromatin condensation at a concentration of 4.00 μg·mL^−1^ [74].

Paclitaxel (PTX), which disrupts microtubule dynamics to inhibit cell division, is another widely used chemotherapeutic. Epithelial cell adhesion molecule (EpCAM) is a transmembrane glycoprotein that is overexpressed in multiple cancers (e.g., breast, ovarian, and colorectal cancers) and is considered an ideal therapeutic target [79]. To improve PTX targeting, Khodadadi et al. constructed an EpCAM-specific DNA aptamer-guided nanosystem (SPIONs@PTX-SYL3C) that selectively delivers PTX to EpCAM-overexpressing breast cancer cells and suppresses proliferation [75]. Research has shown that intercellular adhesion molecule-1 (ICAM-1) is highly expressed in TNBC [80]. Zhu et al. designed a co-delivery nanoparticle platform for PTX and gemcitabine (GEM) and functionalized it with an ICAM-1 ligand to enable targeted drug delivery. The results demonstrated effective accumulation of the nanoparticles in TNBC cells, suggesting a potential new therapeutic approach for metastatic breast cancer [76]. In addition, Chen et al. prepared albumin nanoparticles Nab-PTX-PA based on the Nab^TM^ technology of palmitic acid–paclitaxel (PTX-PA), achieving a high drug-loading system. Nab-PTX-PA not only prolongs retention in tumor tissue but also exhibits superior antitumor activity [77].

Collectively, drug delivery systems leveraging nanotechnology have markedly increased the localized concentration of chemotherapeutic agents within tumor tissues by harnessing the enhanced permeability and retention (EPR) effect and employing active targeting strategies, while simultaneously minimizing off-target toxicity. Through tailored design, these functional nanocarriers improve the physicochemical properties and pharmacokinetic profiles of therapeutic payloads and contribute to overcoming multidrug resistance, thus playing a pivotal role in enhancing both the efficacy and safety of breast cancer chemotherapy.

### 3.2. Immunotherapy and Nanomaterials

Although immune checkpoint inhibitors (ICIs) have achieved breakthroughs in advanced TNBC, their efficacy is often limited by an immunosuppressive “cold” TME, characterized by inadequate T cell infiltration, abundant M2-polarized tumor-associated macrophages (TAMs), and impaired dendritic cell (DC) maturation [81]. To overcome these limitations, nanoparticle-based strategies have been proposed to augment immune checkpoint blockade (Table 3). Exosomes, which are phospholipid bilayer-enclosed vesicles secreted by various cell types, can serve as natural nanocarriers for nucleic acids, proteins, and lipids [82]. Shi et al. engineered exosomes decorated with anti-CD3 and anti-HER2 antibodies to construct a multiplexed antibody-retargeted exosome platform (SMART-Exo) for modulating cellular immunity. This platform effectively activates cytotoxic T cells to target and kill HER2 expressing breast cancer cells, thereby inhibiting tumor growth in HER2 positive models [83].

Cluster of Differentiation 47 (CD47) signaling expressed on the surface of cancer cells mediates immune evasion by engaging the signal regulatory protein α (SIRPα) receptor on macrophages; thus, CD47–SIRPα inhibitors are pursued for tumor immunotherapy [89]. The magnetic nanoparticles (MN) core blocks the CD47–SIRPα pathway while promoting repolarization of TAMs from the M2 to the M1 phenotype, thereby enhancing anti-tumor immunity. Rao et al. developed a cell membrane-coated magnetic nanoparticle (gCM-MN) that blocks CD47–SIRPα interaction and promotes TAM repolarization from M2 to M1 phenotype, thereby enhancing antitumor immunity and suppressing local growth and distant metastasis [86]. In a similar vein, Tang et al. conjugated the DSPE-PEG2000-Mal-RS17 peptide—which specifically binds CD47—to the nanovesicle SPI@hEL, generating SPI@hEL-RS17 nanoparticles that potently inhibit primary tumor growth and prevent metastasis [87,90].

The cyclic guanosine monophosphate–adenosine monophosphate synthase stimulator of interferon genes (cGAS-STING) signaling cascade is a potential target for antitumor immune responses; activation of this pathway can help reverse the locally immunosuppressive TME and enhance the efficacy of immune checkpoint blockade therapy [91,92]. While monophosphoryl lipid A (MPLA) and Mn_3_O_4_ are known STING agonists, their utility is hampered by poor tumor targeting and stability [93,94]. To address this, Liu et al. designed TME-responsive nanoparticles (PMM NPs) co-encapsulating MPLA and Mn_3_O_4_. These NPs not only activate STING signaling but also interact with PD-1 to relieve immunosuppression, leading to potent tumor growth inhibition [88].

Beyond these approaches, emerging forms of regulated cell death such as cuproptosis—a copper-dependent mitochondrial cell death pathway—are being harnessed for immunotherapy. Evidence suggests that cuproptosis can induce immunogenic cell death (ICD), promote DC maturation and cytotoxic T cell infiltration, and remodel the immunosuppressive TME [95]. Nanomedicines associated with anti-programmed cell death protein ligand-1 antibody (αPD-L1) have been demonstrated to enhance immunotherapy for bladder cancer [96,97]. In this work, Zhou et al. constructed a zinc–copper bimetallic nanoplatform (Cu-ZnO_2_@PDA) for TNBC immunotherapy. This system promotes DC maturation and CD8^+^ T cell infiltration via cGAS–STING activation and upregulates PD L1 expression in tumor cells, effectively suppressing TNBC growth and metastasis [85]. In parallel, Li et al. developed a bifunctional CuP/Er nanoplatform that synergizes ferroptosis and cuproptosis to enhance ICI efficacy. Erastin (Er) inhibits system Xc^−^ to deplete glutathione and induce ferroptosis, while the copper component triggers cuproptosis. Together, they induce ICD and upregulate PD L1 expression, significantly inhibiting TNBC growth and preventing brain metastasis [84].

In summary, nanomaterials provide versatile platforms for improving the efficacy of breast cancer immunotherapy. By enabling targeted delivery of immunomodulators, blocking immunosuppressive signals, activating innate immune pathways, and inducing novel forms of immunogenic cell death, nano-enabled strategies can effectively convert immunologically “cold” tumors into “hot” ones, enhancing T cell infiltration and function. This synergistic approach augments the therapeutic effect of immune checkpoint inhibitors and offers new hope for overcoming immunotherapy resistance.

### 3.3. Gene-Therapy and Nanomaterials

Genetic alterations and dysregulated gene expression are closely associated with the initiation and progression of breast cancer, making gene therapy—through correction of defective genes or modulation of gene expression—a promising therapeutic strategy (Table 4). However, the clinical translation of naked nucleic acids is hampered by their poor cellular uptake and instability in systemic circulation. Nanomaterials, with their high loading capacity, scalable production, and controllable release profiles, have thus emerged as attractive vehicles for breast cancer gene therapy [98]. Lipid nanoparticles (LNPs), as non-viral gene delivery systems, are widely used in various disease contexts due to their high payload capacity, low immunogenicity, and cost-effectiveness [99]. In vitro-transcribed mRNA (IVT-mRNA) can express therapeutic antibodies in vivo, enabling the targeting of intracellular drug targets. Rybakova and colleagues developed an LNP-based platform to deliver in vitro-transcribed mRNA (IVT-mRNA) encoding the anti-HER2 antibody trastuzumab. Their system demonstrated enhanced protein expression, prolonged antibody persistence, and significant tumor growth inhibition with improved survival in a murine model, offering a novel strategy for antibody-based breast cancer therapy [100]. High expression of tubulointerstitial nephritis antigen-like 1 (Tinagl1) has been shown to correlate with overall survival in TNBC [101,102]. YAP and TAZ are transcriptional co-activators associated with the Hippo pathway that play crucial roles in breast cancer development and malignant behavior [103,104]. Thus, targeting YAP/TAZ represents a promising therapeutic strategy for breast cancer. CD44 is an important cancer stem cell marker and is increasingly recognized as a valuable target for eradicating invasive tumors [105]. Zhao et al. constructed CD44-specific peptide-modified LNPs for co-delivery of YAP/TAZ siRNA. Compared with non-modified LNPs, these CD44-targeted LNPs exhibited higher cellular uptake and stronger tumor-suppressive effects [106].

The secreted protein Tinagl1 has been identified as a suppressor of TNBC growth via inhibition of EGFR and integrin signaling. Based on this, Musetti et al. designed lipid–protein–DNA–lipid (LPD) nanoparticles loaded with Tinagl1 plasmid DNA (pDNA) to enable localized and targeted Tinagl1 expression. This gene therapy approach led to sustained inhibition of tumor growth and suppression of distant metastasis, highlighting its potential for TNBC treatment [107].

MiRNAs are small non-coding RNAs frequently dysregulated in cancer, leading to either oncogene activation or tumor suppressor silencing. In breast cancer, miR-206 is consistently downregulated, and its restoration has been shown to inhibit malignant proliferation [112,113]. Capitalizing on this tumor-suppressive function, Chaudhari et al. engineered a metallic nanoparticle platform for the efficient intracellular delivery of miR-206. Their data demonstrated that the nanocomplex successfully transported miR-206 into breast cancer cells, induced the cell cycle, and downregulated NOTCH3, thereby triggered apoptosis in breast cancer cells [108]. The tumor-suppressor gene p53 is the most frequently mutated gene in human cancers, with mutations often conferring chemoresistance and poor prognosis [114,115]. Consequently, Strategies to restore wild-type p53 function are therefore of great interest. To improve transfection efficiency, liposomes have been functionalized with targeting ligands to promote receptor-mediated endocytosis [116]. The transferrin receptor (TfR), which is markedly overexpressed on a broad spectrum of cancer cells, has thus been exploited as a docking site for ligand-directed drug delivery [117]. Rejeeth et al. utilized TfR-targeted silica nanoparticles to deliver exogenous p53. Their results demonstrated robust induction of tumor-cell apoptosis and significant suppression of tumor growth in vivo [110].

DNAzymes—catalytic single-stranded DNA molecules that cleave RNA or DNA substrates in the presence of divalent metal ions such as Mn^2+^ or Zn^2+^—have also been explored for cancer gene therapy [118]. Jiang et al. developed a degradable biomimetic nanocapsule incorporating DNAzymes into Mn/Zn–IP6-based cores decorated with small-molecule peptides. This system showed potent antitumor activity and suppressed distal metastasis in both orthotopic and lung metastasis models of breast cancer [109]. In another effort, Yao et al. designed a Zn–Mn ferrite-based nanoheterogeneous composite (DNC-ZMF) integrating cascade DNAzymes and promoter-like elements for combined gene/chemodynamic therapy. The nanocomposite triggers gene therapy by consuming intratumoral protons and glutathione to release metal ions, which in turn drive Fenton-like reactions to generate reactive oxygen species. Together, these mechanisms synergistically inhibited tumor growth, achieving an inhibition rate of 70.4% [111].

In summary, nanomaterials serve as effective non-viral carriers that successfully address key challenges in the systemic delivery of nucleic acid therapeutics, including poor stability, inefficient cellular uptake, and limited targeting specificity. By encapsulating and delivering a variety of gene-based agents—such as siRNA, mRNA, pDNA, miRNA, and DNAzymes—nanosystems enable precise intervention at the genetic level in breast cancer progression. This strategy demonstrates considerable therapeutic potential and is accelerating the translation of gene therapy into clinical practice.

### 3.4. Phototherapy

Phototherapy represents a localized treatment strategy for solid tumors, whose core principle involves the use of light at specific wavelengths to induce photochemical or photothermal effects within the target tissue, thereby selectively eradicating tumor cells [119]. In contrast to conventional treatments, phototherapy offers advantages such as minimal invasiveness and reduced systemic side effects. The two primary forms of phototherapy, PDT and PTT, utilize light in combination with exogenous or endogenous absorbers to generate cytotoxic ROS or elevate local temperature, respectively, achieving therapeutic objectives [120]. Owing to their unique mechanisms of action, both PDT and PTT are frequently employed as adjuvant therapies alongside conventional cancer treatments. Studies have confirmed that these phototherapeutic approaches can effectively overcome chemoresistance, suppress compensatory survival signaling pathways, and enhance drug delivery and accumulation efficiency by modulating the tumor microenvironment, for instance, through improved vascular permeability [121,122]. With advances in nanotechnology, nanoplatforms loaded with PSs or photothermal agents have been developed for breast cancer treatment (Table 5). The following sections will elaborate on PTT, PDT, and their combined strategy (PTT/PDT) in detail.

#### 3.4.1. Photothermal Therapy

PTT is an emerging oncotherapeutic modality that utilizes near-infrared (NIR) light to excite photothermal agents (PTAs), generating localized hyperthermia to ablate neoplastic lesions and induce ICD [143]. PTT is seldom employed as a monotherapy but can significantly enhance the selectivity and efficacy of other treatment modalities when applied in combination. A wide range of nanomaterials—including metallic nanostructures, carbon-based nanosystems, and semiconducting polymers—have been extensively explored as efficient PTAs. Among various PTAs, GNRs are widely employed in oncology due to their high photothermal conversion efficiency and low systemic toxicity [144,145]. Their anisotropic geometry confers strong longitudinal surface plasmon resonance in the NIR region, making them ideal for NIR-triggered PTT [146]. Zhao et al. and Chen et al. independently developed DOX-loaded core–shell nanoplatforms based on GNRs; both studies demonstrated that the combined PTT-chemotherapy regimen produced significantly stronger tumor-suppressive effects than DOX monotherapy, offering a novel therapeutic strategy for breast cancer treatment [123,124]. In another approach, Granja and colleagues co-loaded solid lipid nanoparticles with GNRs and the anticancer drug mitoxantrone. The resulting nanocarriers induced a temperature increase exceeding 20 °C and promoted accelerated mitoxantrone release, significantly enhancing breast cancer cell death [125].

In addition to gold nanoparticles, GO has been widely explored as a photothermal agent owing to its large specific surface area, strong NIR absorption, and good biocompatibility [147]. Li and colleagues developed an ultrafine graphene oxide nanoscale platform (UDP) for DOX delivery, further encapsulating it with polydopamine (PDA). Under NIR irradiation, UDP-treated mice exhibited a rapid temperature increase, significant tumor cell death, and inhibited tumor growth in 4T1-bearing models [126]. Leveraging the excellent photothermal properties of PDA, Lu and colleagues conjugated DOX and ICG via electrostatic interactions and π–π stacking, functionalized the surface with PDA, and attached the conjugate to anaerobic Bifidobacterium (Bif). The resulting Bif@DIP hybrid achieved tumor-selective accumulation and synergistically suppressed tumor growth through combined chemotherapy and PTT [128].

Cell membrane-derived nanoparticles inherit the natural biological properties, enabling immune evasion and tumor-specific targeting [148]. Imiquimod (R837) is an immune adjuvant that promotes DC maturation by activating TLRs and induces macrophage polarization toward the M1 phenotype [149]. Zhang and colleagues designed Fe_3_O_4_ nanoparticles loaded with ICG and R837 using a cell membrane cloaking strategy for combinatorial therapy. The results showed that Fe_3_O_4_ and ICG synergistically produced photothermal effects that enhanced cancer cell ablation, while promoting R837-induced release of tumor-associated antigens and strengthening antitumor immunity [129]. Discoidin domain receptor 2 (DDR2) is a receptor tyrosine kinase aberrantly expressed in breast cancer that promotes cancer-associated fibroblast (CAF) activation and EMT [150,151]. Chen and colleagues developed cancer cell membrane-coated nanospheres (M@P-WIs) for co-delivery of the photothermal agent IR-780 and the DDR2 inhibitor WRG-28. Experimental results demonstrated that M@P-WIs achieved safe and durable clearance of primary tumors and established long-term immune memory, reducing recurrence and metastasis [127].

PTT employs photothermal agents that, upon near-infrared light excitation, generate localized hyperthermia to ablate tumor tissue. Its key advantages include spatiotemporal controllability and minimal invasiveness. Various nanomaterials—such as gold nanorods, graphene oxide, and polydopamine—have been established as efficient photothermal converters. Beyond direct tumor ablation, PTT can induce immunogenic cell death and, when combined with chemotherapy or immunotherapy, exhibits marked synergistic therapeutic effects.

#### 3.4.2. Photodynamic Therapy

PDT exerts its cytotoxic and growth-suppressive effects by activating PSs with laser light at specific wavelengths, generating ROS that disrupt cellular structures and tumor vasculature [152]. The efficacy of this therapy critically depends on key parameters including the PS activation wavelength, irradiation duration, and delivery efficiency of the PSs [153]. Employing nanomaterials or nanocomposites as PS carriers enables selective targeting and enhanced delivery of PSs to tumor tissues, thereby significantly improving the therapeutic outcome of PDT. To overcome the hypoxic TME, Liu et al. engineered a liposomal nanoplatform co-encapsulating nano-platinum (nPt) and the photosensitizer verteporfin (VP). Within the tumor, nPt catalyzes endogenous H_2_O_2_ decomposition to generate O_2_, sustaining VP-mediated PDT; conversely, PDT-induced ROS increase membrane permeability, accelerating nPt release. This reciprocal amplification produced robust cytotoxicity that eradicated primary tumors and suppressed distant metastasis [130]. Porphyrin and its derivatives, as second-generation PSs, absorb at 660–770 nm and accumulate rapidly in cancer cells, attracting broad interest in PDT [154]. Cabral and colleagues designed a nanoemulsion co-loaded with aluminum phthalocyanine and DOX. Upon laser irradiation, this synergistic therapy markedly inhibited tumor proliferation and induced tumor tissue apoptosis, providing a new alternative for the treatment of breast cancer [131]. Besides, Lipid-encapsulated oxygen-loaded nanobubbles (Lipo-NBs-O_2_) were designed to enhance the efficacy of the photosensitizer copper phthalocyanine. Conjugation with anti-HER2 and anti-PD-L1 antibodies yielded DRT@Lipo-PS-NBs-O_2_, which targets HER2-positive breast cancer. Under 808 nm NIR irradiation, the oxygen carried by this system augmented PDT efficacy, increasing the destruction of the primary tumor while effectively inhibiting growth and metastasis of distant tumors [134]. Furthermore, leveraging the specific interaction between lectins and carbohydrates, Calavia et al. functionalized phthalocyanine-coated AuNPs with lactose, enabling precise drug delivery through selective targeting of the overexpressed galactose-binding lectin-1 receptor on breast cancer cells. Experimental results demonstrated that compared with previous studies using other carbohydrates for selective cancer targeting, this functionalized nanoparticle system achieved stronger cytotoxic efficacy with shorter irradiation times and lower radiation doses [155].

Rose bengal (RB), a hydrophilic photosensitizer, exerts anticancer effects by generating singlet oxygen upon irradiation, though its tissue penetration is limited [156,157]. Uddin et al. developed an RB delivery platform using chitosan nanospheres irradiated with 532 nm light. At low doses, it efficiently killed tumor cells while showing no cytotoxicity toward normal mammary epithelial cells [132]. In addition, Wang and colleagues designed a magnetic and pH-responsive nanocomposite, Ce6@MMSN/DOX@FA-PEG-b-PAsp, for co-delivery of the photosensitizer chlorin e6 (Ce6) and DOX. In tumor-bearing mice, the composite exhibited efficient tumor targeting under an external magnetic field. In vitro, DOX release was pH-responsive, effectively reversing tumor cell resistance and inducing apoptosis, thus providing a new platform for breast cancer therapy [133].

Although PDT can directly damage tumor cell DNA and induce apoptosis, its efficacy is limited by cellular DNA repair mechanisms. While, poly(ADP-ribose) polymerase (PARP) facilitates repair of DNA single-strand breaks [158]. To address this, Wu and colleagues developed a multifunctional biomimetic nanoplatform, 4T1Mem@PGA-Ce6/Ola (MPCO), for co-delivery of Ce6 and the PARP inhibitor olaparib (Ola). Experimental results showed that MPCO nanoparticles effectively generate ROS under laser irradiation, damaging tumor cell DNA and inhibiting its repair, thereby suppressing the growth of orthotopic breast tumors and preventing tumor metastasis and recurrence [135].

PDT relies on photosensitizers that, upon irradiation with light of a specific wavelength, generate reactive oxygen species to destroy tumor cells. Nanocarriers significantly improve the targeted delivery, tumor accumulation, and adaptability to hypoxic microenvironments of photosensitizers. By integrating oxygen-generation strategies or DNA repair inhibitors, nanoplatforms further overcome key bottlenecks that limit PDT efficacy, thereby enhancing its antitumor outcomes.

#### 3.4.3. Combined Therapy

Leveraging the complementary mechanisms of action between PDT and PTT, their combined application results in synergistic enhancement of antitumor efficacy. This combination not only facilitates targeted delivery of PSs to tumor tissues but also improves intratumoral ROS accumulation through enhanced local perfusion, thereby achieving synergistic antitumor effects [120]. The realization of this synergistic effect largely relies on the development of multimodal nanoplatforms capable of co-delivering PSs and photothermal agents, which provide crucial technical vehicles for precise implementation of combination therapy. Zero-valent iron (ZVI) exhibits strong reducing capability and Fenton-like catalytic activity. Nano-ZVI (nZVI) retains these catalytic properties while serving as an effective photothermal agent [159,160]. Yu et al. prepared poly(dopamine)-modified nZVI (nZVI@PDA) for breast cancer PTT/PDT. The results demonstrated that, under 808 nm NIR irradiation, nZVI@PDA showed high photothermal conversion efficiency and ROS generation rate, enabling efficient killing of breast cancer cells [136].

Gold nanomaterials, particularly GNRs, are widely studied for PTT-based combination therapy due to their strong tunable LSPR effects and excellent photothermal conversion efficiency [161]. Notably, gold nanostructures can suppress photosensitizer activity prior to reaching the target site, avoiding self-damage and premature activation in circulation, making GNRs ideal for PTT/PDT combinations [139,162]. 5-Animolevulinic acid (ALA), a metabolic precursor of the endogenous photosensitizer protoporphyrin IX (PpIX), is widely used in PDT [163]. Xu et al. constructed a multifunctional nanoplatform by conjugating DOX and ALA to gold nanorod (GNRs-MPH-^ALA/DOX^-PEG) for combined therapy of breast cancer including chemotherapy, PTT, and PDT. Experimental results showed that this platform accumulated effectively in tumor tissue and, under NIR irradiation, generated sufficient ROS for PDT and heat for PTT, leading to complete inhibition of tumor progression (final tumor volume is only 0.43 cm^3^) [137]. In addition, Cheng and colleagues loaded the photosensitizer IR820 and DOX onto organically silicon-coated GNRs, and encapsulated them with hyaluronic acid (HA) to form IR&DOX@NC. This nano-hybrid responded to HAase and glutathione in the TME, enabling preferential accumulation and triggered drug release. Under 808 nm NIR irradiation, it also generated ROS for PDT and induced photothermal effects [138]. In another study, Xu et al. developed a dual-targeted hyaluronic acid-coated gold nanorod platform (GNR-HA-^ALA/Cy7.5^-HER2) targetingHER2 and CD44, which also achieved combined PTT and PDT, and result in complete tumor ablation [139].

In addition to the aforementioned nanomaterials, materials such as polydopamine nanoparticles, albumin nanoparticles, and graphene oxide have also been incorporated into nanoplatforms for multimodal therapy, including PTT/PDT combined with chemotherapy or immunotherapy [140,141,142]. The integration of these therapeutic modalities significantly inhibits tumor growth and mitigates the development of drug resistance, offering a promising comprehensive approach for breast cancer treatment.

The combination of PTT and PDT, or the integration of phototherapy with other treatment modalities such as chemotherapy and immunotherapy, represents a significant direction in cancer therapy. Multifunctional nanoplatforms capable of co-delivering different therapeutic agents enable precise spatiotemporal control over their actions, achieving a synergistic “1 + 1 > 2” therapeutic effect. This approach significantly enhances the inhibitory efficacy against breast cancer, particularly for its refractory subtypes.

## 4. Theranostic Nanomaterials

**Figure 3:**
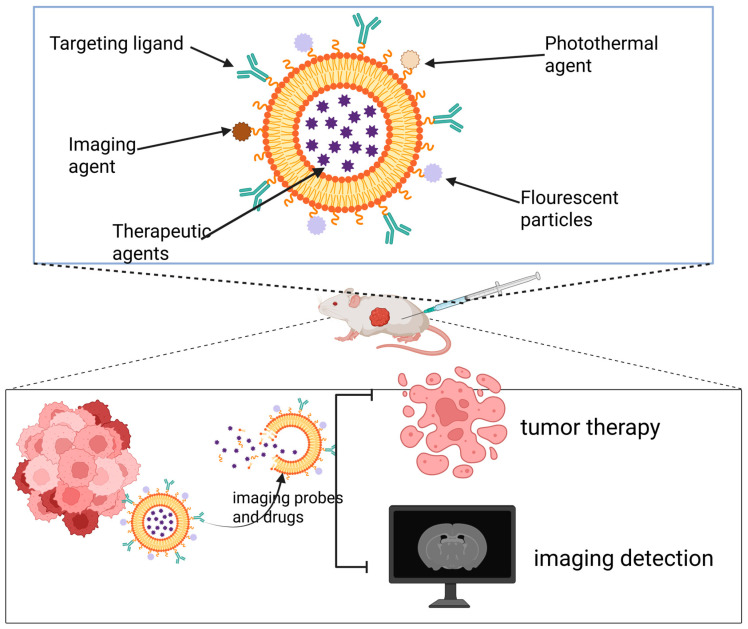
Theranostic nanoplatforms integrate both diagnostic and therapeutic components. By combining multiple imaging modalities with treatment strategies, they enable simultaneous tumor-targeted imaging and multimodal therapy.

Theranostic nanomaterials refer to nanocarrier systems that co-load diagnostic imaging agents and therapeutic drugs within a single nanoparticle, enabling precise disease intervention and real-time monitoring [164]. By incorporating imaging probes, therapeutic agents, or PSs, and engineering them to respond to the TME, these platforms can activate imaging signals and control drug release specifically at the lesion site [165]. This allows for precise matching of therapeutic strategies to the different molecular subtypes of breast cancer. This integrated paradigm of “treatment within diagnosis and monitoring within treatment” significantly enhances the precision, efficiency, and safety of breast cancer diagnosis and therapy, demonstrating the tremendous potential of nanomaterials in advancing personalized precision medicine for breast cancer (Figure 3).

In recent years, Mn -based nanomaterials have shown rapid development in the field of cancer nanodiagnostics and nanotherapeutics. As a naturally occurring element in the human body, Mn participates in physiological processes such as carbohydrate, lipid, and protein metabolism, endowing Mn-containing systems with good biocompatibility and relatively low toxicity [166,167]. Mn-based nanomaterials can significantly shorten T1 and exhibit lower T2 at high magnetic field strengths, enabling their function as T1- and T2-weighted MRI contrast agents [168,169]. Therapeutically, Mn2+ can activate Fenton-like reactions to decompose the elevated hydrogen peroxide in tumors, generating abundant ROS that effectively kill cancer cells [170]. Moreover, Mn^2+^ has been reported to activate the host innate immune system via the cGAS–STING signaling pathway, markedly enhancing DC activation and antigen presentation, and promoting tumor-specific T cell responses, thereby synergistically improving immunotherapy efficacy [171]. These multifaceted properties establish manganese-based nanomaterials as promising agents for integrated cancer theranostics, combining diagnostic imaging, targeted therapy, and immune modulation. Leveraging the multifaceted applications of manganese in cancer imaging and therapy, Zhu et al. developed a manganese-based nanoplatform loaded with glucose oxidase, PTX, and a NIR dye. This platform releases its cargo in acidic environments, not only enhancing T1 contrast in MRI but also achieving efficient inhibition of breast cancer cells, thereby offering a novel diagnostic and therapeutic strategy [172]. Similarly, Ziyaee et al. prepared MnO_2_@Poly (DMAEMA-Co-IA)-MTX NPs based on MnO_2_ nanoparticles, coated with itaconic acid (IA) and conjugated to methotrexate (MTX). In acidic tumor microenvironments, manganese dioxide dissociates into Mn^2+^, thereby enhancing T1 MRI signals. At the same time, Mn dioxide dissociates into Mn^2+^, helping to overcome hypoxia-associated radiotherapy resistance and significantly improving therapeutic efficacy [173]. Biotin, a vitamin receptor commonly overexpressed across various solid tumors, is considered an excellent tumor-targeting moiety for breast cancer treatment. Jain et al. functionalized Mn3O4 nanoparticles with biotin for the targeted delivery of GEM to breast cancer cells. The experimental results show that the resulting nanoparticles constitute a pH-responsive and sustained-release system, providing excellent contrast in both T1- and T2-weighted MRI, and delivering superior therapeutic efficacy compared to free GEM [174].

Gd and iron oxide nanoparticles (Fe_3_O_4_ NPs) are also commonly used MRI contrast agents, producing contrast enhancement in T1- and T2-weighted images, respectively, and are widely applied to cancer imaging. When combined with therapies such as PTT, they enable precise cancer theranostics [175,176]. Mesoporous dopamine (MPDA) nanoparticles function as photothermal transducers and simultaneously serve as anchors for various metal ions, while π–π interactions facilitate the adsorption of the chemotherapy drug like DOX and the anti-inflammatory drug such as MX [177,178,179]. Liu et al. synthesized MX@Arg-Gd-MPDA mesoporous nanoparticles. The chelated Gd^3+^ provides precise T1–T2 bimodal MRI guidance for PTT, while the loaded arginine (Arg) enhances photothermal conversion efficiency and ROS scavenging capability, synergizing with MX to improve the anti-inflammatory effect post-PTT. In a breast cancer mouse model, this system demonstrated notable antitumor efficacy [180]. Chen et al. designed a novel theranostic nanoparticle targeting overexpressed ICAM-1 in TNBC. Gd and DOX were loaded into polyethylene glycol–poly(ε-caprolactone) (PEG-PCL) copolymer-based nanoparticles, functionalized with anti-ICAM-1 antibodies. The results showed effective nanoparticle accumulation at TNBC sites, enhanced T1-weighted MRI contrast, and stronger tumor growth inhibition compared to controls [181]. Oxygen deficiency is a key factor driving invasion, metastasis, and chemotherapy resistance in TNBC. Zhang et al. developed a near-infrared-responsive, on-demand oxygen-releasing nanoplatform (O_2_-PPSiI) loaded with PTX and Gd^3+^, enabling precise and controllable drug release. The platform allows real-time monitoring of its dynamic biodistribution via tumor MRI. Under synergistic NIR irradiation, it suppressed EMT, thereby reducing the migratory and invasive capabilities of TNBC tumors [182]. Magnetic nanogels (MNLs) are a class of nanogels incorporating magnetic nanoparticles, offering advantages such as high stability, remote operability, controllable drug delivery, and MRI capabilities [183]. Zhang and colleagues developed MNLs loaded with Fe_3_O_4_ and DOX, and conjugated with the HER antibody. These MNLs facilitate the release of DOX under acidic conditions, thereby enhancing therapeutic efficacy, while the Fe_3_O_4_ component contributes to an increased contrast in T2-weighted imaging [184].

Photoacoustic imaging (PAI) is an emerging imaging modality that uses optical excitation to generate ultrasound signals, enabling the visualization of hemoglobin concentration and distribution in breast tissue and facilitating malignant tumor identification [185]. Perfluorocarbon (PFC) emulsions hold multifunctional value in diagnosis and therapy, enabling drug delivery, tumor targeting, and imaging with photoacoustic and US modalities [186]. Fernandes and colleagues developed a theranostic PFC nanoemulsion conjugated with silica-coated gold nanoparticles. This nanoemulsion could be loaded with various chemotherapeutic drugs or imaging agents, and selectively induce cancer cell death upon irradiation at 680 nm. Moreover, it exhibited significantly enhanced signal intensity in both US and PAI, with an average ultrasound intensity more than ten times greater than that of whole blood [13]. Carrese et al. developed hybrid albumin-modified nanoparticles with photoacoustic properties, loaded with the anticancer drug DOX. The results indicated that these nanoparticles exhibited a favorable combined chemo- PTT effect, effectively inhibiting breast cancer cell viability, while also possessing strong photoacoustic characteristics (contrast-to-noise ratio: 12–35; signal-to-noise ratio: 13–36). This approach provides a new option for breast cancer theranostics [187]. Phthalocyanines (PC) and naphthalocyanine (NC) dyes have been employed in photoacoustic and ultrasonographic imaging due to their NIR absorption, stability, and imaging contrast [120,188]. They can also serve as alternatives to gold nanoparticles and ICG for PTT [189]. Tian et al. encapsulated various NC and PC dyes and their derivatives in nanoemulsion micelles, and screening revealed that CuNC (Octa)-loaded micelles produced photoacoustic signal intensities tenfold higher than those of gold nanoparticles, while achieving heating performance under laser irradiation comparable to gold nanorods [190].

For patients with early-stage breast cancer, breast-conserving surgery (BCS) is a standard treatment; however, positive tumor margins are strongly associated with local recurrence and distant metastasis [191]. Intraoperative frozen section analysis can assist in assessing margin status but prolongs surgery duration and exhibits low sensitivity (<80%) [192,193]. Therefore, there is an urgent clinical need for a real-time, high-resolution, and highly specific intraoperative margin assessment method. Fluorescence imaging in NIR-II window (1000–1700 nm) offers high sensitivity, superior resolution, and deep tissue penetration, holding great potential for real-time intraoperative imaging [194]. He et al. designed a hafnium (IV)-coordinated NIR-II fluorescent nanoprobe that not only accumulates efficiently at tumor sites but also enables FLI of sub-millimeter-scale microtumors, significantly reducing local tumor recurrence [195]. In addition, Wang et al. developed a nano-system based on ICG and 125I-labeled glycopeptides, which accumulates in tumor tissue under NIR stimulation and enables FLI/SPECR imaging, while enabling precise PTT and PDT upon laser irradiation [196]. Rubtsova et al. also developed a lipid probe for NIR imaging and PDT of TNBC [197].

In conclusion, theranostic nanomaterials mark a pivotal milestone in the management of breast cancer. By integrating diagnostic imaging and therapeutic functions within a single nanosystem, they realize the clinically appealing paradigm of “therapy guided by diagnosis and monitoring enabled by therapy.” Through their responsiveness to the tumor microenvironment and the combination of multiple imaging modalities and treatment approaches, these platforms allow for real-time visualization and precise control over drug delivery, distribution, and efficacy. This integrated capability significantly advances the development of personalized precision medicine for breast cancer and underscores their strong potential for clinical translation (Table 6).

## 5. Clinical Challenges

The clinical translation of nanomaterials in the field of breast cancer therapy has achieved significant milestones (Table 7). Since the 1990s, several nanomedicines have received clinical approval and are currently in use [199]. Leveraging the EPR effect combined with active targeting strategies, nanomedicines significantly enhance localized drug concentration within tumor tissues. Simultaneously, by encapsulating highly toxic chemotherapeutic agents (e.g., liposomal doxorubicin) or utilizing biocompatible carriers (e.g., albumin-bound paclitaxel), they substantially reduce dose-limiting toxicities such as cardiotoxicity and hypersensitivity reactions [98].

Beyond passive accumulation, surface modification with targeting ligands—exemplified by antibody-drug conjugates (ADCs) like trastuzumab emtansine (T-DM1)—enables nanoplatforms to achieve highly precise recognition and eradication of tumor cells with specific molecular phenotypes, thereby markedly improving the specificity of both diagnosis and treatment [204]. Furthermore, nanomaterials demonstrate unique potential in overcoming biological barriers and drug resistance mechanisms. They protect therapeutic payloads from degradation, prolong their circulation half-life, and can reverse or bypass multidrug resistance through co-delivery of multiple agents or incorporation of physical treatment mechanisms (e.g., photothermal effects), offering novel strategies for treating refractory tumors.

Nevertheless, despite these advances and the broad prospects demonstrated in preclinical studies—as detailed in preceding sections—the translation of nanomaterial-based systems into routine clinical practice remains limited. The vast majority of nanomaterial systems reviewed in this article have been validated primarily in in vitro cell cultures and in vivo animal models and tend to complement rather than replace traditional breast cancer management methods. While these models are indispensable for proof-of-concept studies, they cannot fully recapitulate the complexity of human breast cancer. Formidable challenges must be addressed to bridge the gap between laboratory discoveries and clinical applications.

### 5.1. Safety Profile

The biosafety of nanomaterials represents the primary challenge to their clinical translation. Acute risks encompass complement activation-related hypersensitivity reactions, compromised hemocompatibility, and acute hepatosplenic toxicity. Chronic concerns include accumulation in the reticuloendothelial system, gradual leaching of metal ions, and potential genotoxicity [206,207]. A particularly critical issue is immunogenicity: phenomena such as “accelerated blood clearance”—often induced by protein corona formation or intrinsic carrier properties—can substantially undermine both therapeutic efficacy and safety [208]. At present, toxicological data from long-term human exposure remain sparse. Furthermore, species-specific differences in preclinical models and the limited duration of existing evaluations impede accurate prediction of the long-term fate and biological impact of nanomaterials in humans.

### 5.2. Metabolic and Behavioral Controllability

Precise control of the in vivo behavior of nanomedicines is fundamental to achieving efficient and low-toxicity therapy. The EPR effect serves as a core design principle for nanomedicines, providing a pronounced passive targeting advantage by increasing local tumor drug concentration while reducing systemic exposure and associated toxicities. However, the EPR effect exhibits notable limitations that pose formidable challenges for clinical translation. Its manifestation varies markedly across tumor types, patients, and even within different regions of the same tumor [209]. In tumor microenvironments characterized by high interstitial pressure and dense stroma, the uniform diffusion and deep penetration of nanomedicines are severely restricted, compromising therapeutic coverage [210].

Although feasible to track nanomedicine biodistribution using radioactive labeling, multimodal imaging, and other tracing methodologies, actively regulating their metabolic pathways (e.g., balancing hepatic/biliary excretion and renal clearance) and avoiding off-target accumulation remain major unresolved issues [211]. Moreover, nanoparticles undergo dynamic evolution in the complex in vivo milieu, such as the formation and evolving composition of a protein corona, which can substantially alter their presumed biological behavior and complicate accurate prediction and control of their in vivo fate, further increasing clinical unpredictability [208].

### 5.3. Production Costs and Scalability Challenges

The transition from laboratory-scale synthesis to industrial manufacturing constitutes a critical barrier to the commercialization of nanomedicines. The production process faces core issues including complex quality control parameters (e.g., size uniformity, drug loading efficiency, surface modification consistency), significant scale-up effects, and high manufacturing costs [207]. For cutting-edge applications such as central nervous system targeting, the heightened technical barriers and stringent safety requirements further exacerbate the difficulties and costs associated with large-scale production [212]. The absence of unified regulatory evaluation standards also introduces additional uncertainty into their industrialization pathway.

## 6. Conclusions

Nanomaterials, by virtue of their size-dependent effects, ultrahigh surface-to-volume ratio and facile surface engineering, have catalyzed a paradigm shift in breast cancer management. Conventional imaging (mammography, ultrasound) relies predominantly on morphological differences, whereas nano-probes—superparamagnetic iron-oxide nanoparticles for MRI, gold nanorods for CT, quantum dots for NIR-II fluorescence—achieve active tumor accumulation through ligand-mediated targeting (anti-HER2, RGD peptides, etc.), elevating signal-to-noise ratios and lowering detection limits to sub-millimetre lesions. This enables both earlier-stage primary tumors and occult micro-metastases that remain invisible on standard images to be identified, markedly improving diagnostic accuracy.

Concurrently, nanomaterial-based biosensors—graphene field-effect transistors, gold-nanorod SERS chips—allow non-invasive, ultrasensitive detection of circulating tumor DNA, exosomes and protein biomarkers in blood or saliva, extending tissue biopsy to real-time “liquid biopsy” for molecular subtyping, response monitoring and relapse prediction, thereby underpinning precision oncology.

Therapeutically, nanocarriers (liposomes, polymeric micelles) exploit the EPR effect plus active targeting to deliver chemotherapeutics selectively to the tumor bed, overcoming poor solubility, short circulation half-life and off-target toxicities (cardiotoxicity, myelosuppression) inherent to free drugs. Co-delivery of cytotoxics with P-glycoprotein inhibitors or siRNA further circumvents multi-drug resistance. Nanoparticles also function per se as immune adjuvants or as vehicles for tumor antigens/immunomodulators, converting immunologically “cold” tumors into “hot” ones and potentiating immune-checkpoint blockade, thus opening a nano-immunotherapy avenue.

Additionally, nanomaterials serve as ideal agents for PTT and PDT, transducing light into localized heat or ROS to achieve spatiotemporally controlled tumor ablation with minimal collateral damage. Integrating these functionalities into “theranostic” nanoplatforms synchronizes real-time imaging with on-demand treatment, heralding a new era of individualized and precise breast cancer medicine.

## Figures and Tables

**Table 1:** Nanomaterials for Diagnosis of Breast Cancer.

Imaging	Nanosystems	Targeting Molecular	Animal Models/Cell Lines	Outcomes	Reference
MRI	SPION-C595	circulating mucin	MCF-7 cells	produced negative contrast in T2-weighted MRI	[22]
MRI	SPION	RCP	MCF-7 cell line	enhanced T2 contrast ability	[61]
MRI	ZD2-Gd_3_N@C80	EDB-FN	MDA-MB-231 and MCF-7 tumour xenografts mouse models	showed superior r1 and r 2 relaxivities, distinguished between breast cancers of different aggressiveness	[26]
FLI	TQF–Psar	_	4T1 tumor-bearing mice	showed higher NIR-II intensity; displayed a more prolonged blood circulation t1/2 and better tumor accumulation capability	[28]
FLI	R&HV-Gd@ICG	integrin αvβ3 receptor	4T1 tumor-bearing mice	facilitated complete tumor resection, improved RT sensitivity, and reduced mouse tumor progression	[30]
FLI and multispectral optoacoustic tomography (MSOT)	NP-Q-NO_2_	nitroreductase	4T1 tumor-bearing mice	generated significant NIR-I/NIR-II and optoacoustic signals	[62]
CT and FLI	Gd_2_O_3_:Eu^3+^	FR	T-47D or MDA-MB-231 xenograft mouse model	showed intense signals in tumor tissue	[36]

**Table 2:** Chemotherapy Drug Delivery Nanosystem.

Nanocarriers	Drugs	Cell lines	In Vivo Models	Outcomes	Reference
Dox/FA-PLGA-TFA NPs	DOX	_	dimethylbenz[a]anthracene (DMBA)-induced breast cancer mouse model	reduced the side-effects of each single drug, exerted excellent antitumor activity, evaded cancer cell resistance and had a superior safety profile.	[70]
DOX-TPLGA NPs	DOX	MCF-7 and B-Cap-37 cells, and 4T1 cells	4T1 tumor-bearing mice models	exhibited higher drug release under acidic conditions, enhanced uptake by breast cancer cells, and an excellent tumor inhibition rate	[71]
PCL/TA	DOX	MCF-7 cells	_	inhibited tumor cell proliferation, with higher efficacy than free DOX	[72]
PEG-GAx/Pt	CDDP	HeLa, 4 T1, A549, and MCF-7 cells	4 T1 and A549 xenograft tumor models	improved antitumor efficiency with much-reduced toxicity	[73]
CCNP	CDDP	MCF-7 ATCC human breast cancer cells	_	induced early cell death, late apoptosis, and chromatin condensation	[74]
SPIONs@PTX-SYL3C	PTX	MCF-7, 4T1, and MCF-10A cell lines	_	showed a good capacity in recognizing its target cells and inhibiting their growth and division	[75]
GT DcNP	PTX and GEM	4T1 cells, MDA-MB-231 cells, L929 cells, and SK-BR-3 cells	4T1 Lung Metastasis Model	enhanced GEM-PTX exposure and breast cancer suppression	[76]
Nab-PTX-PA	PTX	4T1 cells	4T1 tumor-bearing ICR mice	inhibited tumor cell proliferation, reduced the toxicity of PTX	[77]

**Table 3:** Immunotherapy and Nanomaterials.

Nanocarriers	Targeted Cells	Cell Lines	In Vivo Models	Outcomes	Reference
SMART-Exo	cytotoxic T cells	human PBMCs and HER2^+^ breast cancer cell lines	human HER2^+^ breast cancer xenograft mouse models	inhibited the growth of HER2^+^ breast cancer	[83]
CuP/Er	T cells	triple-negative breast cancer 4T1 cells	4T1 tumor-bearing mice	induced strong immunogenic cell death, enhanced antigen presentation, and upregulated PD-L1 expression	[84]
Cu-ZnO_2_ @PDA	DCs and cytotoxic CD8^+^ T cells	4T1 cells	4T1 orthotopic breast cancer model	promoted DC maturation and cytotoxic CD8+ T cell infiltration; inhibited the growth and metastasis of TNBC	[85]
gCM-MNs	TAMs and CD8^+^ T cells	murine melanoma B16F10 cells and murine mammary carcinoma 4T1 cells	triple negative breast cancer 4T1 tumor model	blocked the CD47-SIRPα pathway and promoted M2 TAM repolarization	[86]
SPI@hEL-RS17	DSc, TAMs and CD4^+^ T helper cells	4T1, B16F10, and A549 tumor cells	4T1 tumor bearing mice	suppressed primary tumor growth, inhibited lung metastasis and prevented tumor recurrence	[87]
PMM NPs	CD4^+^ helper T cells, CD8^+^ cytotoxic T cells, DC and NK cell and TAMs	_	MC38 colon tumor model and low-immunogenic 4T1 breast tumor model	activated the STING signaling pathway and inhibited tumor growth	[88]

**Table 4:** Gene-therapy and Nanomaterials.

Nanocarriers	Targeted Molecule	Cell Lines	In Vivo Models	Outcomes	Reference
LNPs	HER2	SKBR3 and BT-474 cell lines	MDA-MB-23 xenografts tumor models	selectively reduced the volume of HER2-positive tumors and improved animal survival	[100]
LPDs	Tinagl1	4T1 cell line	4T1 tumor-bearing mice models	inhibited tumor growth and the risk of metastasis	[107]
miRNA—PEG—AuNPs	NOTCH 3	MCF-7 cell line		arrested cells in the G0-G1 phase and induced apoptosis	[108]
AKPC-siYT	YAP/TAZ	HCC38 and MDA-MB-231 cell lines	breast tumor cell xenografted zebrafish model	induced robust silencing of YAP/TAZ and enhanced tumor suppression	[106]
PEI-DNAzyme@Mn/Zn-IP6	DNA	MCF-7 human breast cancer cell line	MCF-7 breast tumor mouse model	inhibited tumor growth and distal metastasis	[109]
transferrin–SiNPs–p53	p53	MCF-7 cells	MCF-7 breast tumor mouse model	inhibited tumor growth	[110]
DNC-ZMF	EGR-1 mRNA	MCF-7 cells	MCF-7 tumor-bearing mice	led to a significant therapeutic efficacy of tumor growth suppression	[111]

**Table 5:** Photothermal Therapy and Photodynamic Therapy.

Nanocarriers	Drugs	Cell Lines	In Vivo Models	Outcomes	Reference
AuNR@NCMC/DOX	DOX	MCF-7 cell line	MCF-7 tumor-bearing mice	higher tumor accumulation of DOX and AuNR and stronger inhibition of tumor growth	[123]
Au@mPDA	DOX	4T1 cell line	4T1 tumor-bearing mice	improved the anti-tumor effects	[124]
SLNs	mitoxantrone	MCF-7 cell line	_	enhanced breast cancer cell death	[125]
UDP	DOX	4T1 cell line	4T1 tumor-bearing mice	induced tumor cell death and inhibited tumor growth	[126]
M@P-Wis	WRG-28	EO771 cell line	EO771 orthotopic breast cancer model	achieved complete primary tumor regression and established long-term immunological memory	[127]
Bif@DIP	DOX	4T1, MCF-7 cell lines	4T1 tumor-bearing mice	showed good photothermal conversion efficiency; resulted in effective and sustained anti-tumor effect	[128]
RIFe@TRM	R837	4T1 cell line	4T1 tumor-bearing mice	demonstrated outstanding synergy in chemodynamic/immunotherapy/photothermal therapies; strengthened antitumor immunotherapy	[129]
nano-Pt/VP@Mlipo	Pt	4T1 cell line	orthotopic 4T1 breast tumor mouse model	inhibited the growth of aggressive 4T1 tumors and their lung metastasis	[130]
NE-PcDoxo	DOX		4T1 tumor-bearing mice	increased tissue necrosis and massive decrease in proliferative cells	[131]
RB-encapsulated nanoparticles	_	MCF-7 and PC3 cell lines	_	inhibited the tumor growth	[132]
Ce6@MMSN/DOX@FA-PEG-b-Pasp	DOX	MCF-7/ADR cell line	MCF-7/ADR tumor bearing mice	reversed resistance and induced apoptosis of breast cancer cells	[133]
DRT@Lipo-PS-NBs-O2	_	HER2^+^ 4T1 cell line	HER2^+^ 4T1 tumor bearing mice	increased the destruction of the primary and inhibited the growth and metastasis of distant tumors	[134]
MPCO	Ola	4T1 cell line	4T1 primary tumor-bearing mice model	suppressed the growth of orthotopic breast tumors and prevented tumor metastasis and recurrence	[135]
nZVI@PDA	_	MCF-7 cell line	_	offered high light-to-heat conversion and ROS generation efficiency	[136]
GNRs-MPH-^ALA/DOX^-PEG	DOX	MCF-7 cell line	MCF-7 tumor-bearing mice model	more efficiently killed MCF-7 cells and completely suppress tumor growth without obvious systemic toxicity	[137]
IR&DOX@NC	DOX	4T1 cell line	4T1 tumor-bearing mice model	allowed the efficient release of loaded DOX and IR 820 in tumor sites; triggered the generation of reactive oxygen species and produced remarkable photothermal efficacy	[138]
GNR-HA-^ALA/Cy7.5^-HER2	_	HER2-positive MCF-7 cell line	HER2-positive MCF-7 tumor-bearing mice model	enhanced the cellular uptake; efficiently generated reactive ROS and heat; completely eliminated tumor tissues	[139]
SPoD NP	_	MCF-7 and 4T1 cell lines	4T1 tumor-bearing mice model	inhibited both normoxic and hypoxic cancer cell growth; prevented lung metastasis and splenomegaly	[140]
IR780-ZnS@HSA	_	MDA-MB-231 and 4T1 cell lines	4T1 tumor-bearing mice model	inhibited tumor growth and improved the efficacy of aPD-L1	[141]
IR780/SB/DOPA-rGO	_	MCF-7 cell line	_	decreased breast cancer cells’ viability	[142]

**Table 6:** Theranostic nanomaterials.

Imaging	Drugs	Nanosystems	Animal Models/Cell Lines	Outcomes	Reference
MRI	MTX	MnO_2_@Poly(DMAEMA-Co-IA)-MTX NPs	MCF-10A, MCF-7, and 4T1 cells	inhibited MCF-7 cell viability more effectively and displayed pH-responsive contrast enhancement	[173]
MRI	GEM	GEM-loaded Biotin-PEG@MNCs	MD-MBA-231 human breast cancer cells	exhibited significantly higher cell growth inhibition, showed both longitudinal and transverse relaxivity about 0.091 and 7.66 mM-1 s-1 at 3.0 T MRI scanner	[174]
MRI	PTX	O_2_-PPSiI	MDA-MB-231 orthotopic xenografts	enhanced T1 positive contrast, alleviate tumor hypoxia, inhibited the process of EMT	[182]
MRI	MX	MX@Arg-Gd-MPDA	4T1 xenograft mouse model	enabled dual-mode T1/T2 imaging of tumor sites, suppressed tumor growth	[180]
MRI/FLI	PTX	NanoMn-GOx-PTX	4T1 tumor-bearing mouse model	enhanced T1 contrast in MRI, induce the natural immune response and oxidative damage of tumor tissue	[172]
MRI	DOX	Gd-DOX@PEG/PCL	MDA-MB-231 tumor-bearing mouse model	enhanced T1 contrast ability, suppressed tumor growth	[181]
MRI	DOX	NIPAM-AA-MAPEG	MCF-7 tumor-bearing mouse model	enhanced T2 contrast ability, accumulate in HER2^+^ breast cancer tissues	[184]
MRI	PTX	O_2_-PPSiI	MDA-MB-231 orthotopic xenografts	alleviated tumor hypoxia and inhibited the process of EMT	[182]
PAI/US	DOX/5-FU	PFH-NEs-scAuNPs	MCF-7 cell line	enhanced contrast imaging of tumors and served as a drug-delivery vehicle for therapeutic purposes	[13]
PAI/US	DOX	MelaSil_Ag-HSA@DOX	Hs578T cell line	resulted a shorter exposure time to doxorubicin and a lower drug dose; showed a detectable signal increasing with NP concentration	[187]
PA/US	_	CuNC(Octa)-loaded micelles	4T1 tumor bearing mice model	demonstrate efficient heat generation with PTT and a strong PA signal with a peak absorbance at ~870 nm	[190]
FLI/SPECT	_	ICG@ADY (^125^I) NPs	4T1 tumor bearing mice model	presented highly sensitive FLI/SPECT images to realize cancer diagnosis; accurate PTT/PDT	[196]
FLI/MRI	_	HA-ICG-Fe-PDA	TNBC-xenografted mice	produced enhanced magnetic resonance signal, allowed the activatable NIR fluorescence imaging, achieved PTT/PDT and photothermal imaging	[198]
FLI	DTSPO	GSH	4T1 tumor-bearing mice	the fluorescence signal intensity could persist for up to 144 h post-injection, identified the submillimeter-sized primary and residual tumors	[195]

**Table 7:** Nanomedicines approved for clinical use for breast cancer treatment.

Nanocarrier	Drug	Indications	Approve	Nanocarrier	Reference
Protein nanoparticle	PTX	metastatic breast cancer and pancreatic cancer, advanced NSCLC	United States of America (USA) (2005), Europe (2008)	Protein nanoparticle	[200]
Protein micelle	PTX	metastatic or advanced breast cancer, ovarian cancer, NSCLC	India (2006)	Protein micelle	[201]
Liposome	PTX	solid tumors, including breast cancer, ovarian cancer and non-small cell lung cancer	China (2003)	Liposome	[199]
Liposome	DOX citrate	metastatic breast cancer	Europe and Canada (2000)	Liposome	[202]
Liposome	DOX hydrochloride	metastatic breast cancer, Kaposi’s sarcoma, ovarian cancer and multiple myeloma	USA (1995), Europe (1996)	Liposome	[203]
Antibody-drug conjugate	Maytansinoid	HER2-positive metastatic breast cancer	USA (2013)	Antibody-drug conjugate	[204]
Antibody-drug conjugate	SN-38	patients with metastatic TNBC who received at least two prior therapies for metastatic disease	USA (2020)	Antibody-drug conjugate	[205]

## Data Availability

No new data were created or analyzed in this study.

## Abbreviations

The following abbreviations are used in this manuscript:

AC	Doxorubicin/Cyclophosphamide
ALA	5-Aminolevulinic Acid
AuNPs	Gold Nanoparticles
BCS	Breast-Conserving Surgery
CA15-3	Carbohydrate Antigen 15-3
CAF	Cancer-Associated Fibroblast
CEA	Carcinoembryonic Antigen
CD47	Cluster of Differentiation 47
CDDP	Cisplatin
cGAS-STING	Cyclic Guanosine Monophosphate–Adenosine Monophosphate Synthase–Stimulator of Interferon Genes
CT	Computed Tomography
ctDNA	Circulating Tumor DNA
DCs	Dendritic Cells
DDR2	Discoidin Domain Receptor 2
DOX	Doxorubicin
EDB-FN	Extra domain-B Fibronectin
EMT	Epithelial–Mesenchymal Transition
EpCAM	Epithelial Cell Adhesion Molecule
EPR	Enhanced Permeability and Retention
ER	Estrogen Receptor
EVs	Extracellular Vesicles
FLI	Fluorescence Imaging
Gd^3+^	Gadolinium(III)
GEM	Gemcitabine
GNRs	Gold Nanorods
GO	Graphene Oxide
IARC	International Agency for Research on Cancer
ICAM-1	Intercellular Adhesion Molecule-1
ICD	Immunogenic Cell Death
ICIs	Immune Checkpoint Inhibitors
ICG	Indocyanine Green
ELISA	Enzyme-Linked Immunosorbent Assay
IVT-mRNA	In Vitro-Transcribed mRNA
LNPs	Lipid Nanoparticles
LSPR	Localized Surface Plasmon Resonance
MNLs	Magnetic Nanogels
Mn^2+^	Manganese (II)
MOFs	Metal–Organic Frameworks
MPLA	Monophosphoryl Lipid A
MRI	Magnetic Resonance Imaging
MX	Meloxicam
miRNAs	microRNAs
nZVI	Nano-Zero-Valent Iron
NC	Naphthalocyanine
NIR-II	Second Near-Infrared Window
Ola	Olaparib
PAI	Photoacoustic Imaging
PARP	Poly(ADP-Ribose) Polymerase
PC	Phthalocyanines
PCL	Polycaprolactone
PD-L1	Programmed Death-Ligand 1
PDA	Polydopamine
PDT	Photodynamic Therapy
PLGA	Poly(lactic-co-glycolic acid)
PFC	Perfluorocarbon
PEG	Polyethylene Glycol
PTT	Photothermal Therapy
PTX	Paclitaxel
PpIX	Protoporphyrin IX
PSs	Photosensitizers
RB	Rose Bengal
R837	Imiquimod
RCP	Riboflavin Carrier Protein
Rf	Riboflavin
ROS	Reactive Oxygen Species
SIRPα	Signal Regulatory Protein α
SPECT	Single-Photon Emission Computed Tomography
SPIONs	Superparamagnetic Iron Oxide Nanoparticles
TAMs	Tumor-Associated Macrophages
TFA	Trans-Ferulic Acid
Tinagl1	Tubulointerstitial Nephritis Antigen-Like 1
TLRs	Toll-Like Receptors
TME	Tumor Microenvironment
TfR	Transferrin Receptor
US	Ultrasound
VP	Verteporfin
HER2	Human Epidermal Growth Factor Receptor 2
Ce6	Chlorin e6
MTX	Methotrexate
MSOT	Multispectral Optoacoustic Tomography
MR	Mannose Receptor
Con A	Concanavalin A
ADCs	antibody-drug conjugates
USA	United States of America

## Glossary

Biomarker	A measurable biological molecule (e.g., protein, gene, miRNA) found in tissues or body fluids that indicates the presence, status, or progression of a disease such as cancer. Examples in breast cancer include HER2, CA15-3, and ctDNA.
Enhanced Permeability and Retention Effect	A phenomenon whereby nanoparticles of a certain size range tend to accumulate preferentially in tumor tissues due to the leaky nature of tumor blood vessels and impaired lymphatic drainage, forming the basis for passive targeting in nanomedicine.
Immunogenic Cell Death	A form of regulated cell death that activates the adaptive immune system. Dying cells release danger signals and tumor-associated antigens, which can stimulate dendritic cell maturation and T-cell responses, turning the tumor microenvironment from “cold” to “hot.”
Liquid Biopsy	A non-invasive diagnostic technique that analyzes circulating biomarkers (e.g., ctDNA, extracellular vesicles, circulating tumor cells) in body fluids like blood to obtain real-time information about tumor genetics, molecular profile, and treatment response, overcoming limitations of traditional tissue biopsies.
Nanoprobe	A nanoscale agent engineered for diagnostic imaging. It typically consists of a nanomaterial core conjugated with targeting ligands and imaging labels (e.g., fluorescent dyes, contrast agents) to enable sensitive and specific detection of biological targets or pathological processes.
Photodynamic Therapy	A treatment modality that uses light of a specific wavelength to activate a photosensitizer drug localized in tumor tissue. The activated photosensitizer generates cytotoxic ROS, leading to selective destruction of cancer cells and damage to tumor vasculature.
Photothermal Therapy	A therapeutic approach that employs photothermal agents (e.g., gold nanorods, graphene oxide) to convert absorbed near-infrared light energy into localized heat, thereby inducing hyperthermia and ablation of tumor cells.
Theranostics	An integrated approach that combines diagnostic imaging and therapeutic capabilities within a single nanoplatform. It enables real-time monitoring of drug delivery, treatment response assessment, and image-guided therapy, aiming for personalized and precision medicine.
